# Performance of a deep-learning algorithm for referable thoracic abnormalities on chest radiographs: A multicenter study of a health screening cohort

**DOI:** 10.1371/journal.pone.0246472

**Published:** 2021-02-19

**Authors:** Eun Young Kim, Young Jae Kim, Won-Jun Choi, Gi Pyo Lee, Ye Ra Choi, Kwang Nam Jin, Young Jun Cho

**Affiliations:** 1 Department of Radiology, Gil Medical Center, Gachon University College of Medicine, Incheon, South Korea; 2 Department of Biomedical Engineering, Gachon University College of Medicine, Incheon, South Korea; 3 Department of Occupational and Environmental Medicine, Gachon University College of Medicine, Incheon, South Korea; 4 Department of Radiology, Boramae Medical Center, Seoul, South Korea; 5 Seoul National University College of Medicine, Seoul, South Korea; 6 Department of Radiology, Konyang University Hospital, Daejeon, South Korea; 7 Konyang University School of Medicine, Daejeon, South Korea; Fondazione Istituto G.Giglio di Cefalu, ITALY

## Abstract

**Purpose:**

This study evaluated the performance of a commercially available deep-learning algorithm (DLA) (Insight CXR, Lunit, Seoul, South Korea) for referable thoracic abnormalities on chest X-ray (CXR) using a consecutively collected multicenter health screening cohort.

**Methods and materials:**

A consecutive health screening cohort of participants who underwent both CXR and chest computed tomography (CT) within 1 month was retrospectively collected from three institutions’ health care clinics (n = 5,887). Referable thoracic abnormalities were defined as any radiologic findings requiring further diagnostic evaluation or management, including DLA-target lesions of nodule/mass, consolidation, or pneumothorax. We evaluated the diagnostic performance of the DLA for referable thoracic abnormalities using the area under the receiver operating characteristic (ROC) curve (AUC), sensitivity, and specificity using ground truth based on chest CT (CT-GT). In addition, for CT-GT-positive cases, three independent radiologist readings were performed on CXR and clear visible (when more than two radiologists called) and visible (at least one radiologist called) abnormalities were defined as CXR-GTs (clear visible CXR-GT and visible CXR-GT, respectively) to evaluate the performance of the DLA.

**Results:**

Among 5,887 subjects (4,329 males; mean age 54±11 years), referable thoracic abnormalities were found in 618 (10.5%) based on CT-GT. DLA-target lesions were observed in 223 (4.0%), nodule/mass in 202 (3.4%), consolidation in 31 (0.5%), pneumothorax in one 1 (<0.1%), and DLA-non-target lesions in 409 (6.9%). For referable thoracic abnormalities based on CT-GT, the DLA showed an AUC of 0.771 (95% confidence interval [CI], 0.751–0.791), a sensitivity of 69.6%, and a specificity of 74.0%. Based on CXR-GT, the prevalence of referable thoracic abnormalities decreased, with visible and clear visible abnormalities found in 405 (6.9%) and 227 (3.9%) cases, respectively. The performance of the DLA increased significantly when using CXR-GTs, with an AUC of 0.839 (95% CI, 0.829–0.848), a sensitivity of 82.7%, and s specificity of 73.2% based on visible CXR-GT and an AUC of 0.872 (95% CI, 0.863–0.880, *P* <0.001 for the AUC comparison of GT-CT vs. clear visible CXR-GT), a sensitivity of 83.3%, and a specificity of 78.8% based on clear visible CXR-GT.

**Conclusion:**

The DLA provided fair-to-good stand-alone performance for the detection of referable thoracic abnormalities in a multicenter consecutive health screening cohort. The DLA showed varied performance according to the different methods of ground truth.

## Introduction

Chest X-ray (CXR) can assist in the diagnosis and management of cardiothoracic disorders; however, in asymptomatic outpatients or the general population, CXR has limited benefit, leading to additional unnecessary examinations with risks of additional harm and costs. In a cohort study of primary care outpatients who received a CXR despite the absence of respiratory symptoms, only 1.2% of CXR detected a major abnormality and 93% of these findings proved to be false positives and none required treatment on further inspection [1].

Nonetheless, CXR is widely used as a component of periodic health examinations for asymptomatic outpatients or the general population because the examination has many advantages in terms of easy accessibility, low cost, and negligible radiation exposure. In Korea, the National Health Service has offered a free CXR screening biennially to all residents aged 40 years or older [2]. Furthermore, CXR has been widely performed for pre-employment and pre-military service medical screening.

However, the interpretation of CXRs is subject to human error and depends on reader expertise. Approximately 20% of errors in diagnostic radiology occurred during the interpretation of radiography, half of which were related to CXR [3]. The low diagnostic yield and substantial inter- and intra-reader variability remain persistent weaknesses of CXR as a screening tool. However, for CXR to become an effective screening tool for an asymptomatic general population with a low pre-test probability for chest disease, the method needs to show high sensitivity and low false-positive results. The limitations of human expert-based diagnosis have provided a strong motivation for the use of computer technology to improve the speed and accuracy of the diagnostic process. Recent advances in deep-learning algorithms (DLA) are expected to improve the diagnostic performance for the screening of lung cancer, pneumonia, and pulmonary tuberculosis on CXR [4–10].

The purpose of the present study was to evaluate the standalone performance of a commercially available DLA for thoracic abnormalities on CXR in a consecutively collected multicenter health screening cohort.

## Materials and methods

This retrospective cohort study was approved by the institutional review boards of three participating institutions (approval number: GFIRB2019-175 for Gil Medical Center, 10-2019-48 for Boramae Medical Center, 2019-05-022 for Konyang University Hospital). All data were de-identified and the requirement for written informed consent was waived. Lunit in Seoul, Korea, provided corporate support to build an image annotation tool. None of the authors have any financial interests or conflicts of interest with the industry or the product used in this study. The authors maintained full control of the data. We present the following article in accordance with the Strengthening the Reporting of Observational Studies in Epidemiology (STROBE) reporting checklist (S1 Appendix).

### Study population for the diagnostic cohort study

**Fig 1:**
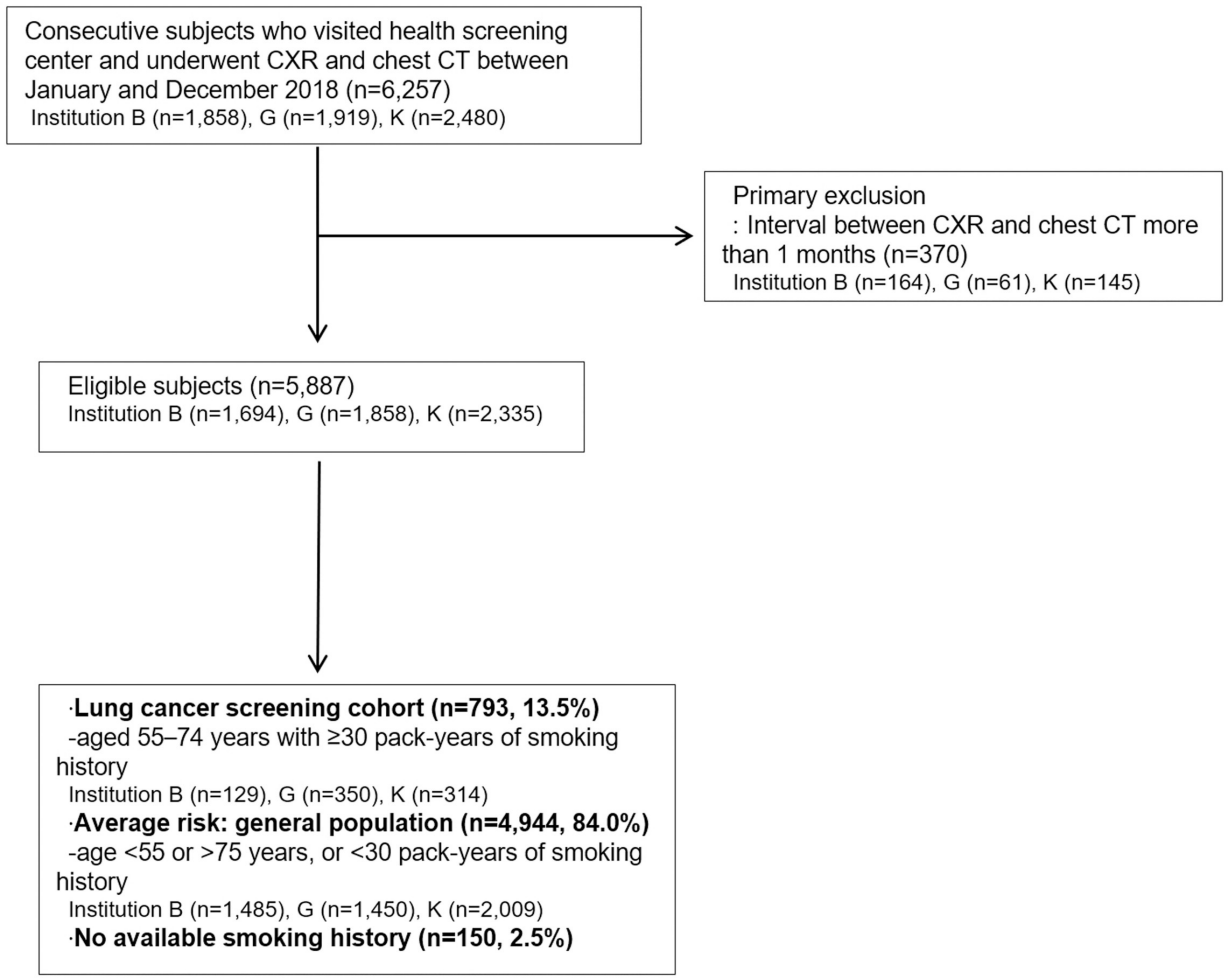
Flow chart of the study population.

Data from a total of 5,887 consecutive subjects who visited the health screening center of the three institutions and underwent CXR and chest CT in 2018 were retrospectively investigated from the radiology database and medical records system. Subjects who underwent chest CT from CXR with intervals of 1 month or more were excluded. Data on age, sex, smoking history (pack-years), exam date of CXR, and chest CT were retrospectively collected. Based on age and smoking history, the cohort was classified as have a high risk of lung cancer (aged 55–74 years with ≥30 pack-years of smoking history) or an average risk (general population). Fig 1 shows the flow chart of the study population.

### DLA for chest radiographs

We used a commercially available DLA (Lunit INSIGHT for Chest Radiography Version 2.5.7.4; Lunit, Seoul, South Korea) approved by the Korean Ministry of Food and Drug Safety. This version of DLA was developed for the detection of three major radiologic findings (the target lesion types are nodule/mass, consolidation, and pneumothorax) using a deep convolutional neural network [7]. Further detailed information about its development and validation is presented in S1 Fig. DLA-detected thoracic lesions are marked as a color map with abnormality score (%). The abnormality score indicates the probability value (0–100%) that the CXR contains malignant nodule/mass, consolidation, or pneumothorax. We used a predefined cutoff value of 15%, as it showed high sensitivity (95%) in the internal validation dataset [11].

### Reference standards for referable thoracic abnormalities

After the de-identification of all CXR, the images were uploaded and annotated for ground truth (GT) using a customized web-based labeling tool provided by Lunit. With labeled GT, the system automatically classified the DLA results as true-positive when there was overlap of at least one pixel with the GT; otherwise, the lesion was classified as false-positive or false-negative.

The reference standard for referable thoracic abnormalities on CXR was determined by three adjudicators (C.Y.J., J.K.N. K.E.Y., with 19, 13, and 12 years of experience in thoracic imaging, respectively), primarily based on the findings of the nearest chest CT. They also reviewed follow-up CXR images and medical records to determine the clinical diagnosis.

Referable thoracic abnormalities, defined as any CXR findings requiring further diagnostic evaluation or management, were classified into 10 lesion types and the lesions were annotated as a box region of interest (ROI). They included three DLA-target lesion types (nodule/mass, consolidation, and pneumothorax) and seven DLA-non-target lesion types (atelectasis or fibrosis, bronchiectasis, cardiomegaly, diffuse interstitial lung opacities, mediastinal lesion, pleural effusion, and others). These imaging findings were adapted and partially modified from the labeling standards of the ChestX-ray14 or MIMIC-CXR databases [8, 12] and the Fleischner Society glossary of terms for thoracic imaging [13]. Furthermore, final clinical diagnoses were categorized based on the 10^th^ edition of the International Classification of Diseases (ICD)-10 [14] or radiologic descriptions for thoracic lesions [13].

The original GT was made based on chest CT, which is considered the most precise method as a reference standard for CXR. However, CT-based GT (CT-GT) is not practical and does not reflect real-world clinical situations. CXR examinations infrequently accompany chest CT examinations; CT examination is performed for suspicious or ambiguous CXR findings for which further evaluation is needed under clinical suspicion. Furthermore, when the adjudicators annotated referable thoracic abnormalities on CXR based on retrospective inspection of chest CT findings, very subtle lesions were labeled on CXR, which were difficult to identify on CXR without CT guidance. To overcome this limitation, we established additional GTs based on consensus CXR readings. For cases with any referable thoracic abnormalities on the original CT-GT, we asked three radiologists (K.R.H, S.Y.S, and H.S.H with 7, 10, and 13 years of experience in thoracic radiology, respectively) to evaluate the existence of referable thoracic abnormalities on the CXR. Finally, we made subsequent GTs based on consensus CXR readings (CXR-GTs); namely, clear visible CXR-GT (for more than two calls) and visible CXR-GT (for at least one call).

### Statistical analysis

The results are presented as percentages for categorical variables and as means (± standard deviation) for continuous variables. Primarily, we evaluated the diagnostic performance of the DLA for referable thoracic abnormalities based on CT-GT, in terms of the area under the receiver operating characteristic (ROC) curve (AUC), sensitivity, specificity, positive predictive value (precision), negative predictive value, and F1 score (the harmonic mean of precision and recall). To evaluate lesion-wise localization performance, area under the alternative free-response ROC curves (AUAFROCs) were used as performance measures of jackknife alternative free-response ROC (JAFROC), the curve was plotted with the lesion localization fraction (LLF) against the probability of at least one false-positive (FP) per normal CXR. The total number of false-positive markings divided by the total number of CXRs was defined as the number of false-positive markings per image (FPPI). In addition, true detection rate (number of correctly localized lesions/the total number of lesions) was also evaluated. Finally, we evaluated the performance of the DLA using CXR-GTs (clear visible and visible CXR-GTs). To assess AUC differences when evaluating the DLA using different reference standard methods, we used either the paired or unpaired versions of DeLong’s test for ROC curves, as appropriate. Statistical analyses were performed using MedCalc version 19.5.1 or R version 3.5.3.

In the case of multiple testing, pairwise comparison and post-hoc analysis were performed, and *P-*values and 95% confidence intervals (CIs) were corrected using Bonferroni’s method. *P-*values less than 0.05 were considered to indicate significant differences.

## Results

### Baseline characteristics and lesion types of the referable thoracic abnormalities

Table 1 shows the demographic features of the study subjects (4,329 males and 1,558 females; mean age, 54±11 years). A total of 618 (10.5%) subjects had referable thoracic abnormalities, including: nodule/mass (n = 202, 3.4%), consolidation (n = 31, 0.5%), pneumothorax (n = 1, <0.1%), and DLA-non-target abnormalities (n = 409, 6.9%), respectively (Table 2).

**Table 1:** Demographic description of the dataset.

	Institutions			Total	*P*-value
	B	G	K		
	(n = 1,694)	(n = 1,858)	(n = 2,335)	(n = 5,887)	
Sex, men	996 (58.8)	1,458 (78.5)	1,875 (80.3)	4,329 (73.5)	<0.001
Age (years)	56±11	53±11	54±13	54±11	<0.001
Non-smoker	876 (51.7)	621 (33.4)	742 (31.9)	2239 (38.1)	<0.001
Ex-smoker	415 (24.5)	431 (23.2)	576 (24.8)	1422 (24.2)	
Current smoker	403 (23.8)	805 (43.3)	1007 (43.3)	2215 (37.7)	
High risk of lung cancer^†^	129 (8.0)	350 (19.4)	314 (13.5)	793 (13.8)	<0.001
Average risk of lung cancer	1,485 (92.0)	1,450 (80.6)	2,009 (86.5)	4,944 (86.2)	

Note: Except where indicated, data are mean (± SD) or number (%). SD = standard deviation missing data for pack-year information (n = 150, 2.5%). Comparisons of means and proportions between institutions for demographic information were performed using analysis of variance (ANOVA) and chi-squared tests.

^†^High-risk lung cancer indicates age 55–74 years with a smoking history of 30 pack-years or more

**Table 2:** Lesion types of referable thoracic abnormalities on chest radiographs (determined based on computed tomography [CT]).

Lesion type	Institutions				*P*-value*
	B	G	K	Total	
	(n = 1,694)	(n = 1,858)	(n = 2,335)	(n = 5,887)	
Normal (no referable thoracic abnormality)	1,571	1,594	2,105	5,270	<0.001
	(92.7)	(85.8)	(90.1)	(89.5)	
Target lesions^†^	50 (3.0)	68 (3.7)	115 (4.9)	233 (4.0)	0.003
Nodule/mass	44(2.6)	65 (3.5)	94 (4.0)	203 (3.4)	
Consolidation	6 (0.4)	3 (0.2)	23 (1.0)	32 (0.5)	
Pneumothorax	0 (0.0)	0 (0.0)	1 (<0.1)	1 (<0.1)	
Non-target lesions	74 (4.4)	205 (11.0)	127 (5.4)	406 (6.9)	<0.001
Atelectasis or fibrosis	18 (1.1)	21 (1.1)	22 (0.9)	61 (1.0)	
Bronchiectasis	35 (2.1)	70 (3.8)	21 (0.9)	126 (2.1)	
Cardiomegaly	5 (0.2)	10 (0.5)	28 (1.2)	43 (0.7)	
Diffuse interstitial lung opacities	4 (0.2)	6 (0.3)	10 (0.4)	20 (0.3)	
Mediastinal lesion	1 (0.1)	0 (0.0)	3 (0.1)	4 (0.1)	
Pleural effusion	1 (0.1)	1 (0.1)	1 (0.1)	2 (0.1)	
Other	13 (0.8)	101 (5.4)	46 (2.0)	160 (2.7)	
Sum of target or non-target lesions	127	277	250	654	<0.001
No. of lesion type per subject^‡^	0.07 (0–2)	0.15 (0–2)	0.11 (0–2)	0.11 (0–2)	

Note: Except where indicated, data are numbers of patients, with percentages in parentheses.

*Comparison of proportions between institutions for each lesion type by chi-squared tests.

^†^Target lesions were dedicated lesion types for the deep-learning algorithm used in this study.

^‡^No. of lesion types per subject was calculated in subjects with target or non-target lesions.

The numbers in parentheses are ranges.

**Fig 2:**
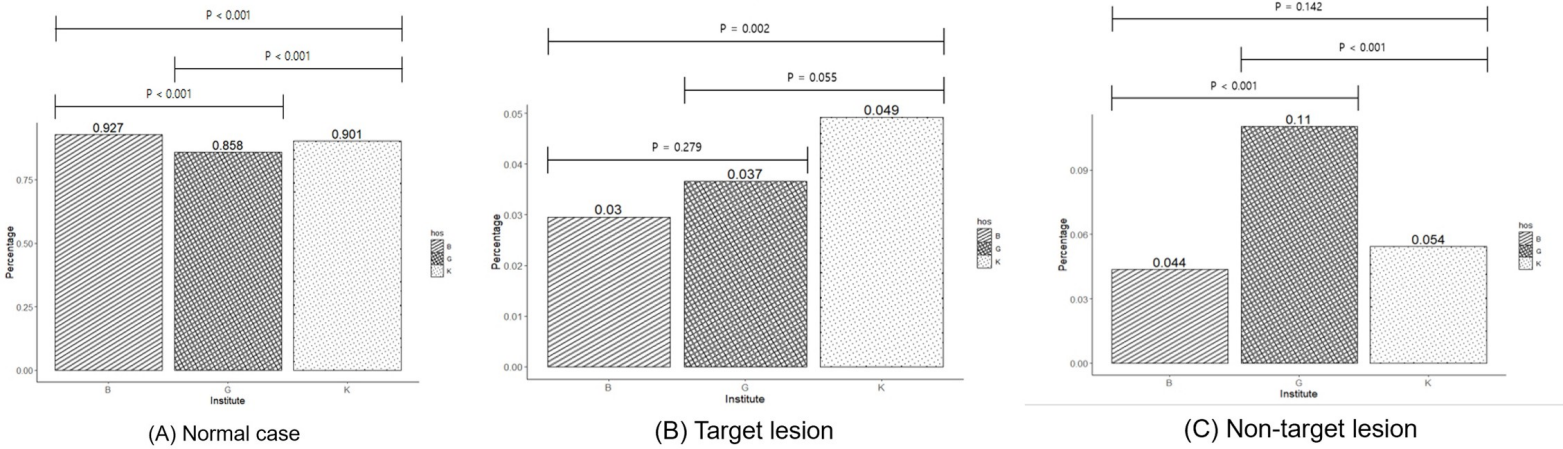
The prevalence of normal cases, and target and non-target lesions of a deep-learning algorithm (DLA) showing significant differences between the three institutions. Institution G has fewer normal cases and more DLA-non-target lesions compared to those of the other two institutions.

The normal cases differed significantly among the three institutions (Bonferroni-corrected *Ps* <0.001); the prevalence of normal cases was lowest at institution G (85.8%), and followed by institution K (90.1%), and institution B (92.7%). Furthermore, the proportions of target and non-target lesions also differed significantly, in which institution B had fewer target-lesions compared to those in institution K (B vs. K; 3% vs. 4.9% Bonferroni-corrected *P =* 0.002*)* and institution G had more non-target lesions compared to those in the other two institutions (G vs. K: 11% vs. 4.4% and G vs. B: 11% vs. 5.4%, Bonferroni-corrected *Ps <* 0.001*)* (Fig 2).

Regarding categorized clinical diagnoses, benign pulmonary nodules were the most common (n = 183, 3.1%), while infection and malignant neoplasm occurred in 61 (1.0%) and 24 (0.4%) patients, respectively (S1 Table).

### Standalone performance of DLA based on CT-GT

To classify the presence of any referable thoracic abnormalities (yes/no) based on the CT-GT, the overall diagnostic performance of the DLA was as follows: AUC of 0.77 (95% CI, 0. 76–0.78), sensitivity of 69.6% (95% CI, 65.8–73.2%), and specificity of 74.0% (95% CI, 72.8–75.1%) (Table 3). For lesion-wise localization, the AUAFROC was 0.65 (95% CI, 0.64, 0.67) and FPPI and true detection rate was 0.384 and 0.481, respectively (S2 Table).

**Table 3:** Standalone performance of the deep-learning algorithm (DLA) for visible referable thoracic abnormalities on chest radiographs in the multicenter health screening cohort based on chest computed tomography (CT) findings.

		Performance of DLA					
Institution	Reference standard	AUC	Sensitivity	Specificity	PPV	NPV	F1 score (%)
			(%)	(%)	(%)	(%)	
B (n = 1,694)	CT-GT	0.74	63.93	74.55	16.32	96.38	26.00
		(0.72, 0.76)					
	Visible CXR-GT	0.88	88.71	74.08	11.51	99.42	20.37
		(0.86, 0.89)					
	Clear visible CXR-GT	0.91	94.59	73.26	7.32	99.84	13.59
		(0.89–0.92)					
G (n = 1,858)	CT-GT	0.71	75.00	56.52	22.22	93.17	34.29
		(0.69, 0.74)					
	Visible CXR-GT	0.74	79.12	55.43	16.16	96.07	26.84
		(0.72, 0.76)					
	Clear visible CXR-GT	0.75	83.16	53.94	8.87	98.35	16.02
		(0.73–0.77					
K (n = 2,335)	CT-GT	0.84	66.81	85.88	34.29	95.91	45.32
		(0.82, 0.85)					
	Visible CXR-GT	0.91	84.47	85.46	30.09	98.67	30.09
		(0.90, 0.92)					
	Clear visible CXR-GT	0.95	94.74	83.84	19.91	99.73	32.91
		(0.94–0.96)					
Total (n = 5,887)	CT-GT	0.77	69.74	73.62	23.67	95.40	35.34
		(0.76, 0.78)					
	Visible CXR-GT	0.84	82.72	72.89	18.40	98.28	30.10
		(0.83, 0.85)					
	Clear visible CXR-GT	0.87	89.87	71.43	11.20	99.43	19.92
		(0.86–0.88)					

Note: Numbers in parentheses are 95% CI. AUC = Area under the receiver operator characteristic curve, CI = confidence interval, CT-GT = ground truth based on chest computed tomography (CT), CXR-GT = ground truth based on chest X-ray; PPV, positive predictive value; NPV, negative predictive value

### Performance evaluation using different reference standards

Among cases with referable thoracic abnormalities (n = 618) primarily based on CT-GT, three radiologists independently performed subsequent evaluations for the presence of visible referable thoracic abnormalities on CXR. On consensus CXR reading (CXR-GTs), the prevalence of referable thoracic abnormalities decreased, compared to 618 (10.5%) CT-GT-positive cases, visible (visible CXR-GT), and clear visible (clear visible CXR-GT) abnormalities were found in 405 (6.9%) and 227 (3.9%) cases, respectively.

Based on the CXR-GTs, the performance of the DLA increased, with an AUC of 0.84 (95% CI, 0.83–0.85), sensitivity of 82.7% (95% CI, 78.7–86.3%), and specificity of 73.2% (95% CI, 72.0–74.4%) based on visible CXR-GT and an AUC of 0.87 (95% CI, 0.86–0.88), sensitivity of 83.3% (95% CI, 77.8–87.9%), and specificity of 78.8% (95% CI, 77.7–79.9%) based on clear visible CXR-GT. Comparison of AUCs showed that the overall performance of the DLA was significantly better when using clear visible CXR-GT than CT-GT as a reference standard (AUC: 0.87 vs. 0.77, *P* <0.001) (Fig 1).

**Fig 3:**
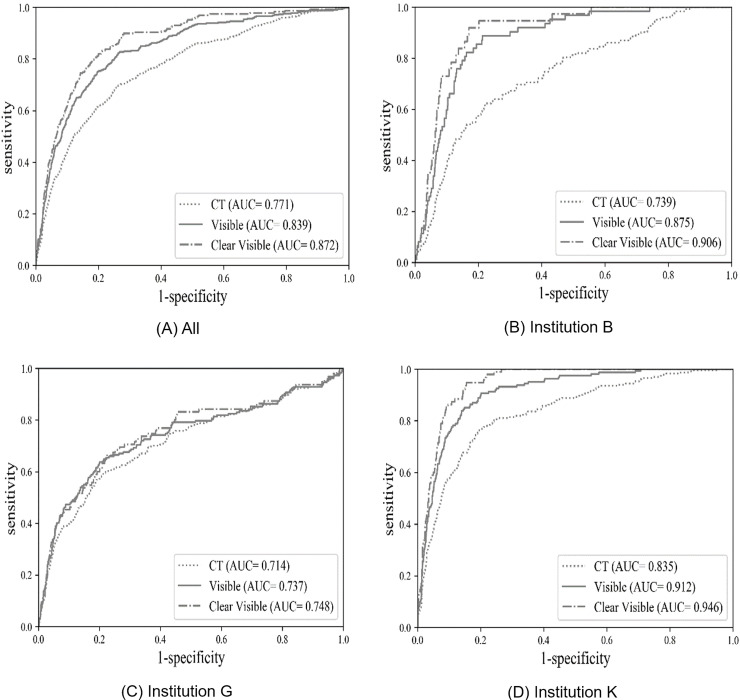
Receiver operating characteristic curve (ROC) curve of a deep-learning algorithm (DLA) for referable thoracic abnormalities on chest radiography based on different standard reference methods. The area under the ROC curve (AUC) shows better performance when using visible and clear visible CXR compared to using CT as ground truth methods, except for institution G (C).

Two institutions (B and K) showed significantly better performance when using CXR-GTs compared to CT-GT. However, institution G did not show significantly better performance when using clear visible GT (Fig 3).

## Discussion

This study evaluated the standalone performance of a commercial DLA for CXR using a consecutively collected multicenter health screening cohort. In the health screening cohort, we can expect a low prevalence of chest disorder compared to an inpatient or outpatient cohort with symptoms and risk factors for respiratory disorder. In the low pre-test probability setting, CXR needs to show high sensitivity and low false-positive results to become an effective screening tool for the asymptomatic general population. Therefore, we selected a threshold of 0.16 because the primary purpose of screening lies in sensitively detecting thoracic abnormalities, including early lung cancer and tuberculosis.

Our study results showed fair to good diagnostic performance of the DLA for CXR and revealed significantly different performance results for different reference standard methods. Based on CT-GT, the performance of the DLA for referable thoracic abnormalities was fair. However, the performance increased significantly when CXR-GTs were used, with the DLA showing the best performance based on clear visible CXR-GT. On CT-GT, subtle lesions were included as abnormalities as compared to CXR-GTs. Among cases with referable thoracic abnormalities (n = 618, 10.5%) primarily based on chest CT, visible and clear visible abnormalities decreased the number of patients with referable thoracic abnormalities to 405 (6.9%) and 227 (3.9%) on consensus CXR reading, respectively. When the adjudicators annotated abnormalities originally based on CT, they inevitably tended to call very subtle lesions that are difficult to detect on prospective inspection on CXR.

Interestingly, the performance did not increase significantly in one institution that had a higher number of non-target lesions compared to those in the other institutions. The prevalence of lesion types is dependent on the clinical setting (inpatient, outpatient, emergency room, and health care clinic) and hospital level (tertiary academic hospitals: institution G and K; secondary general hospital: institution B) and location (institution G and K were located in Incheon and Daejeon in Korea, respectively, while institution B is located in the capital city of Korea, Seoul). The institution G showed the lowest AUC when evaluated using CT-GT and the performance improvement was not observed after using subsequent CXR-GTs. In deep-learning modeling, the DLA is trained to detect and classify using a training dataset. Although some overlap was present between the imaging findings of DLA-target and DLA-non-target lesions as increased opacity, the lesion types that were not included in the initial training process (DLA-non-target lesions) did not show good performance. Therefore, the interpretation of DLA results requires care as the performance of the DLA could depend on the disease prevalence and lesion characteristics (target and non-target lesion distribution) as well as the standard reference methods.

In previous studies, DLA for CXR showed excellent performance, similar to the expert radiologist reading for the diagnosis of lung cancer, tuberculosis, and multiple abnormal findings [7, 11, 15]. These studies used previous version of DLA of slight different DLA architectures, and the evaluation were conducted on experimentally designed datasets with prepared cases of lung cancer, tuberculosis, and normal, which have either one abnormal finding or pure normal cases. While these studies confirmed the technical validity of DLAs, in the real-world setting, the incidence of the disease differs between clinical settings and mixed abnormal findings of DLA-target and non-target lesions are common. Furthermore, image quality and comorbidities are the obstacles to DLA-based diagnosis from CXR. Therefore, the performance evaluation of DLA in a consecutively collected cohort in a real clinical situation is important to prove the clinical validity of this approach. Distinct from previous version, the DLA used in the present study does not use the lung segmentation module and the baseline architecture has been changed to ResNet34 [16]. Attend-and-Compare Module was used in the intermediate layers to improve detection performance [17] and AutoAugment algorithm [18] combined with conventional image processing techniques such as brightness, contrast adjustment, blurring, and random cropping were applied to augment the training dataset. Furthermore, the final layer output four different abnormality-specific channels (mass/nodule, pneumothorax, consolidation, and abnormalities), each representing the probability map for the corresponding abnormality (S1 Fig). To verify differences in diagnostic capabilities according to DLA architecture differences, further investigation with different DLAs using the diagnostic cohort is needed.

Our study has several limitations. First, subjects who underwent only CXR without chest CT in health clinics were excluded which may lead to selection bias. Most of the subjects who visited the health clinics did not undergo chest CT. Second, the performance of the DLA was evaluated using a specific version of a commercial product with a predefined cut-off value set for high sensitivity. Therefore, the results were obtained under certain circumstances and care is required in interpreting the results of the DLA for other products or other clinical settings. Third, the results of our study are limited to one country, so the generalizability to racial differences in other countries is uncertain.

In conclusion, the results of the present study demonstrated the overall fair to good stand-alone performance to determine the presence of referable thoracic abnormalities in a multicenter consecutive health screening cohort. The DLA showed varying performance depending on the type of reference standard method and the frequency of specific lesion types.

## Supporting information

S1 Appendix STROBE statement—checklist of items that should be included in reports of observational studies. (DOC)

S1 Fig Architecture of the deep-learning algorithm. (DOCX)

S1 Table Clinical diagnoses of the multicenter health screening cohort. (DOCX)

S2 Table The lesion-wise performance of deep-learning algorithm. (DOCX)
