# Correction: Performance of a deep-learning algorithm for referable thoracic abnormalities on chest radiographs: A multicenter study of a health screening cohort

**DOI:** 10.1371/journal.pone.0251045

**Published:** 2021-04-28

**Authors:** Eun Young Kim, Young Jae Kim, Won-Jun Choi, Gi Pyo Lee, Ye Ra Choi, Kwang Nam Jin, Young Jun Cho

There are errors in the Competing Interests statement. The correct Competing Interests statement is as follows: KNJ has received research grant funding from Lunit Inc. for activities not related to the present article. This does not alter our adherence to PLOS ONE policies on sharing data and materials. Other authors have no potential conflicts of interest to disclose.
