# Supplementary material for: Performance of a deep-learning algorithm for referable thoracic abnormalities on chest radiographs: A multicenter study of a health screening cohort
Source: PLoS One. 2021 Feb 19;16(2):e0246472. doi: 10.1371/journal.pone.0246472 (PMC7894861; doi:10.1371/journal.pone.0246472)
Supplement: S1 Fig — (DOCX) [file pone.0246472.s002.docx]

S1 Fig. Architecture of the deep-learning algorithm


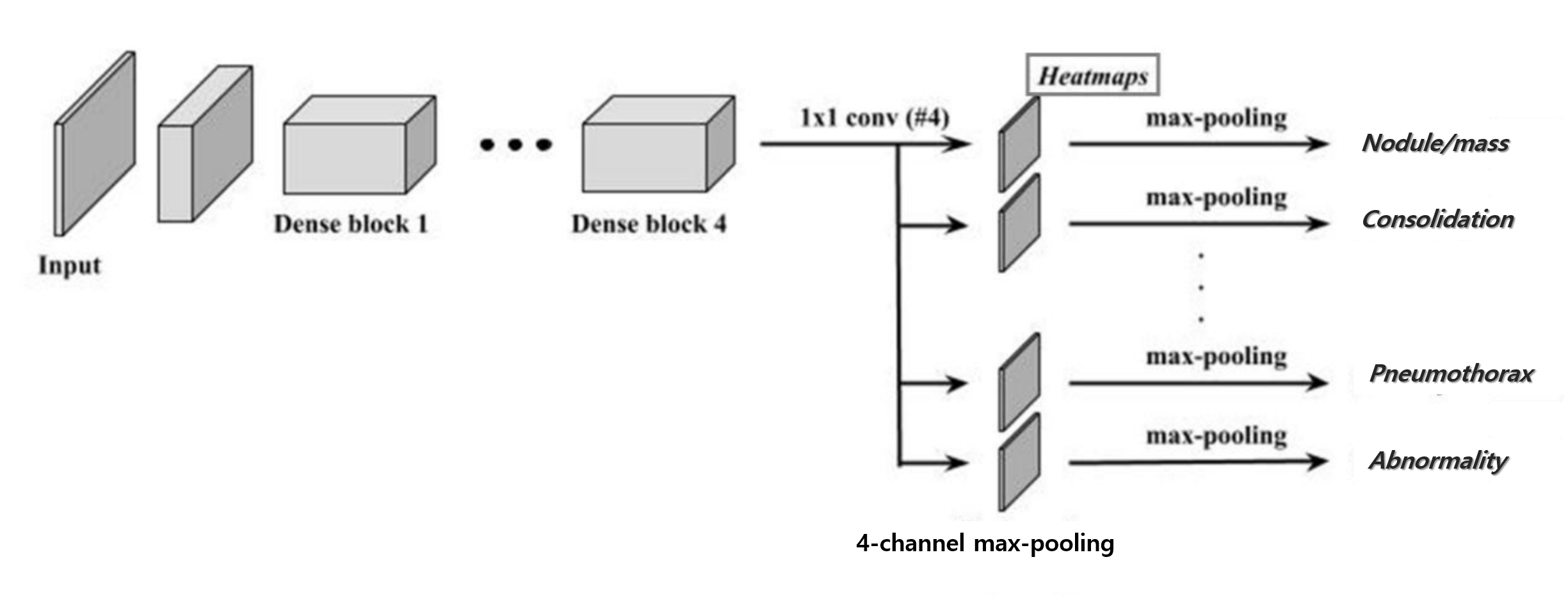


ResNet-34 based architecture of Lunit INSIGHT CXR. Raw pixel map of the DICOM images are passed through 34 convolutional layers that serve as the feature extractor projecting the chest radiograph onto a good representation space. This is followed by four 1 by 1 convolution heads which creates heatmaps of each of four findings. Pixel-wise binary cross entropy loss and image-level binary cross entropy loss are used during training.
