# Supplementary material for: Performance of a deep-learning algorithm for referable thoracic abnormalities on chest radiographs: A multicenter study of a health screening cohort
Source: PLoS One. 2021 Feb 19;16(2):e0246472. doi: 10.1371/journal.pone.0246472 (PMC7894861; doi:10.1371/journal.pone.0246472)
Supplement: S1 Table — (DOCX) [file pone.0246472.s003.docx]

S1 Table**.** Clinical diagnoses in the multicenter health screening cohort

|  | Institutions | | | Total  (n=5,887) |
| --- | --- | --- | --- | --- |
|  | B  (n=1,694) | G  (n=1,858) | K  (n=2,335) |  |
| **Normal** | 1,575 (93.0) | 1,526 (82.1) | 2,055 (88.0) | 5,156 (87.6) |
| **Pulmonary parenchymal diseases** | | | | |
| Infection | 11 (0.6) | 14 (0.8) | 36 (1.5) | 61 (1.0) |
| Malignant neoplasm of the bronchus and lung | 5 (0.3) | 8 (0.4) | 9 (0.4) | 22 (0.4) |
| Secondary malignant neoplasm of the lung | 0 | 2 (0.1) | 0 (0.0) | 2 (<0.1) |
| Benign pulmonary nodule | 19 (1.1) | 52 (2.8) | 112 (4.8) | 183 (3.1) |
| Pulmonary nodules of indeterminate nature | 11 (0.6) | 14 (0.8) | 7 (0.3) | 32 (0.5) |
| Bronchiectasis | 35 (2.1) | 90 (4.8) | 20 (0.9) | 145 (2.5) |
| Atelectasis or pulmonary fibrosis | 19 (1.1) | 49 (2.6) | 19 (0.8) | 87 (1.5) |
| Diffuse interstitial lung disease | 2 (0.1) | 6 (0.3) | 10 (0.4) | 18 (0.3) |
| Pneumoconiosis | 0 | 2 (0.1) | 2 (0.1) | 4 (0.1) |
| Severe emphysema | 0 (0.0) | 10 (0.5) | 18 (0.8) | 28 (0.5) |
| Pulmonary parenchymal opacities, NOS | 7 (0.4) | 5 (0.3) | 4 (0.2) | 16 (0.3) |
| Pulmonary vascular malformation or other congenital lung lesions | 1 (0.1) | 1 (0.1) | 0 (0.0) | 2 (<0.1) |
| **Pleural diseases** | | | | |
| Pleural effusion without parenchymal abnormalities | 0 (0.0) | 5 (0.3) | 3 (0.1) | 8 (0.1) |
| Chronic pleural lesion or pleural tumor | 2 (0.1) | 26 (1.4) | 6 (0.3) | 34 (0.6) |
| Pneumothorax | 0 (0.0) | 0 (0.0) | 1 (<0.1) | 1 (<0.1) |
| **Mediastinal diseases** | | | | |
| Cardiomegaly or pericardial effusion | 5 (0.3) | 13 (0.7) | 25 (1.1) | 43 (0.7) |
| Mediastinal mass | 1 (0.1) | 3 (0.2) | 2 (0.1) | 6 (0.1) |
| **Bone and other** | | | | |
| Rib fracture, benign rib lesions, or bone metastasis | 1 (0.1) | 26 (1.4) | 1 (<0.1) | 28 (0.5) |
| Aorta or pulmonary vascular lesion | 0 (0.0) | 4 (0.1) | 2 (0.1) | 6 (0.1) |
| Other | 0 (0.0) | 2 (0.1) | 3 (0.1) | 5 (0.1) |

Note: Data are provided as numbers of patients, with percentages in parentheses; NOS = not otherwise specified, N/A = not applicable.
