# Supplementary material for: Performance of a deep-learning algorithm for referable thoracic abnormalities on chest radiographs: A multicenter study of a health screening cohort
Source: PLoS One. 2021 Feb 19;16(2):e0246472. doi: 10.1371/journal.pone.0246472 (PMC7894861; doi:10.1371/journal.pone.0246472)
Supplement: S2 Table — (DOCX) [file pone.0246472.s004.docx]

S2 Table. The lesion-wise performance of deep-learning algorithm

| GT-CT | Institution | | | Total  (n=5,887) |
| --- | --- | --- | --- | --- |
|  | B (n=1,694) | G (n=1,858) | K (n=2,335) |  |
| AUAFROC | 0.667  (0.631, 0.707) | 0.561 (0.531, 0.582) | 0.713 (0.683, 0.747) | 0.652 (0.637, 0.670) |
| False positive per image | 0.355 | 0.667 | 0.180 | 0.384 |
| True detection rate | 0.421 | 0.451 | 0.539 | 0.481 |
